# Dissemination of an American Indian Culturally Centered Community-Based Participatory Research Family Listening Program: Implications for Global Indigenous Well-Being

**DOI:** 10.3390/genealogy4040099

**Published:** 2020-09-30

**Authors:** Lorenda Belone, Rebecca Rae, Katherine A. Hirchak, Benelda Cohoe-Belone, Ardena Orosco, Kevin Shendo, Nina Wallerstein

**Affiliations:** 1 College of Education and Human Sciences, Health, Exercise & Sports Sciences Department, University of New Mexico, Albuquerque, NM 87131, USA; 2 Center for Participatory Research, College of Population Health, University of New Mexico, Albuquerque, NM 87131, USA; 3 Elson S. Floyd College of Medicine, Washington State University, 412 E. Spokane Falls Blvd, Spokane, WA 99202, USA; 4 Ramah Navajo Health Promotions Program, Ramah Navajo School Board, Inc., Pinehill, NM 87357, USA; 5 Mescalero Apache Prevention Program, Mescalero Apache Tribe, Mescalero, NM 88340, USA; 6 Jemez Education Department, Pueblo of Jemez, Jemez Pueblo, NM 87024, USA

**Keywords:** indigenous, American Indian, culture-centered, community-based participatory research, Family Listening Program, dissemination and implementation, tribal research team, tribal communities

## Abstract

We introduce a culture-centered indigenous program called the Family Listening Program (FLP), which was developed through a long-standing community-based participatory research (CBPR) partnership involving tribal research teams (TRTs) from three American Indian communities (Apache, Navajo, and Pueblo) with the University of New Mexico’s Center for Participatory Research (UNM-CPR). This paper provides background information on the TRT/UNM-CPR multi-generational FLP intervention funded by the National Institute on Drug Abuse and how it is poised to take the next steps of dissemination and implementation (D&I). In preparing for the next steps, the TRT/UNM-CPR team piloted two FLP dissemination activities, first at the state-level and then nationally; this paper describes these activities. Based on the learnings from the pilot dissemination, the TRT/UNM-CPR team developed an innovative D&I model by integrating a community-based participatory research culture-centered science (CBPR-CCS) approach with the Interactive Systems Framework (ISF) to examine the uptake, cultural acceptance, and sustainability of the FLP as an evidence-based indigenous family program.

## 1. Introduction

The indigenous Family Listening Program (FLP) is a long-term participatory research partnership between the University of New Mexico’s Center for Participatory Research (UNM-CPR) and three southwest American Indian tribal communities of Jemez Pueblo, Mescalero Apache, and Ramah Navajo. Embracing community-based participatory research (CBPR) principles (Israel et al. 2013) and culture-centered approaches (Dutta 2007; Wallerstein et al. 2019), the FLP’s three American Indian Tribal-specific family curricula were co-created, piloted, and implemented by tribal research teams (TRT) with three different National Institutes of Health (NIH) grant cycles over the past 14 years. Each curriculum is centered within each community’s tribal histories, language, knowledge, and practices (Belone et al. 2012, 2016, 2017) and is administered over 12 two-hour evening family sessions. As a dinner-based intergenerational prevention program for fourth/fifth graders, parents, and elders, the FLP is currently being rigorously evaluated to assess reduced child risk behaviors and strengthened protective factors of connections to culture, history, and language.

The Pueblo of Jemez, traditionally known as Hemish of Walatowa, is located in north central New Mexico, has ~3400 members, and is the only Towa-speaking community, with language fluency surveyed in 2006 at 80–85%. With community members now concerned about a decline in the Towa language, language preservation (with Head Start immersion), and Hemish ways of life are tribal priorities, with the belief that cultural preservation enhances health and educational attainment (Shendo et al. 2012). Since 1999, Jemez has partnered with UNM-CPR in multiple research studies; their Family Circle Program was created and piloted with NIH Native American Research Centers for Health (NARCH) III (2005–2009) funding (Belone et al. 2016).

The Ramah Band of Navajo, with ~3000 members, is located in western New Mexico and is one of three noncontiguous reservations from the main Navajo Nation. Ramah Navajo was the first New Mexico tribe to exercise self-determination for education in the 1970s, with oversight from the Ramah Navajo School Board, Inc. (RNSB). In 1978, RNSB expanded its role to oversee health and social services. Since 2000, Ramah Navajo has partnered with UNM-CPR in multiple research studies; their FLP was created and piloted with NARCH III (2005–2009) funding (Belone et al. 2012).

The Mescalero Apache Tribe has over 5000 enrolled members in south-central New Mexico, with three sub-bands, Mescalero, Chiricahua, and Lipan Apache, speaking Southern Athabaskan, though with much language loss. Mescalero has been a leader in native sovereignty, including business and water rights. With healthcare delivery under the Indian Health Services, their FLP was the first tribal-directed prevention research program, working with UNM-CPR with NARCH V (2009–2014) funding (Belone et al. 2017).

Based on the success of piloting three culture-centered family curricula, the three TRTs agreed to collaborate in the rigorous testing of each program and to aggregate child and parent data to build an evidence base of the FLP through current NIH-National Institute on Drug Abuse (NIDA) funding (2014–2021). Though still in effectiveness trial, other southwest tribal communities have expressed an interest in learning more about the FLP, and with promising preliminary data from the effectiveness trial, the UNM-TRT team began to actively plan and disseminate information about the program, including the processes of building a tribal team and using a CBPR approach to culturally re-center and implement the family program with new tribal communities. This paper will describe the pilot dissemination efforts by the TRT/UNM-CPR partnership and their experience in conducting a state-level and national dissemination workshop.

### 1.1. Family Listening Program Curriculum

The FLP is a two-hour dinner program with 12 sessions that uses cultural connectedness, empowerment, and a positive youth development model that is led in each tribe’s language or bilingually with a focus on communication skills, New Mexico health education standards, and drug/alcohol prevention messages with reinforced cultural traditions and indigenous knowledge. Research has found evidence that the connection to history, land, language, traditional food, and culture can have a positive impact on indigenous health (King et al. 2009; McIvor et al. 2009; Whitbeck et al. 2004). There are also findings that increased cultural connectedness can have a positive impact on academic success for children between the fifth and eighth grade (Whitbeck et al. 2001a, 2001b).

Each curriculum covers evidence-based and cognitive-behavioral exercises that have been re-centered culturally within each community. The first five sessions are grounded in indigenous teachings specific to each tribe: A *Welcoming Dinner*—emphasizing cultural foods and eating together as a family; *My Family*—relationships, core values, and cultural roles of families; *Our Tribal History*—communities’ ancestral lineage of the people; *Our Tribal Way of Life*—phases of life, cultural roles, and responsibilities; and *Community Vision*—strengths and visions for each community. The next seven sessions focus on cognitive-behavioral exercises: *Community Challenges*—challenges and solutions needed; *Communication and Help Seeking*—cultural norms and behaviors of extended support systems; *Recognizing/Managing Types of Anger*—approaching anger from traditional ways; *Problem Solving Strategies/Being Different*—exploring discrimination while highlighting beauty in differences; and *Positive Relationships* of respect and support. By session six, the families start their *Community Action Projects* (CAPs) supporting empowerment efforts. Sessions end with *Building Social Support* and *Making a Commitment*—with a sharing of CAPs. All sessions are based on a cyclical reflection process, based on Brazilian Educator Paulo Freire’s emancipatory educational methodology, which embodies deep listening to each other, engaging in dialogue across generations, and supporting the families to engage in actions for change, which then repeats in subsequent sessions of re-listening, dialogue, action, and reflection (Freire 1970).

In our current FLP NIDA-funded study (2014–2021), we have recruited parent–child dyads from our three tribal communities, resulting in over 560 participants [program (n = 295) and comparison (n = 265)]. Measures for children included assessment of depression, anxiety, problem-solving skills, cultural participation, alcohol and drug use, and other factors. Adult measures included parenting skill, coping strategies, community support, and engagement in traditional and cultural activities.

### 1.2. Dissemination and Implementation Science

Dissemination and implementation (D&I) science has become increasingly important as health interventions are developed, tested, and found to be effective in improving health outcomes in a specific setting, only to fail to be integrated into larger practice (Battaglia and Glasgow 2018; Brownson et al. 2015). Implementation of research findings in the lab to clinics and other settings have been known to take a 17-year period (Drolet and Lorenzi 2011; Fishbein and Ridenour 2013; Rabin and Brownson 2012). Other implementation challenges in translating evidence from one setting to diverse contexts are equally important. Sandler (2007) has critiqued the need to maintain “evidence-based fidelity”, as a structural injustice, as this fidelity does not confront power imbalances when academic interventions are brought into community settings, without questioning whether they are appropriate for adoption to the local context. Further complicating matters, past D&I efforts have not adequately considered the importance of sovereignty, culture, and needs of American Indian/Alaska Native (AI/AN) Nations. A requirement to conduct research among many AI/AN tribal members includes obtaining legal approvals from the governing bodies of AI/AN Nations (e.g., tribal resolutions, tribal institutional review boards). Some tribal council resolutions have gone so far as to entirely dismiss outsider empirically derived solutions. Given the health inequities experienced by AI/AN communities, a rapid approach to improve not only health programs, but also health outcomes, is needed. Processes to address barriers and facilitators to implementing interventions at the individual, organization, and policy levels is a necessary next step (Crump et al. 2017; Ferlie and Shortell 2001).

The concept of culture-centeredness deepens an approach to culture beyond a set of beliefs or superficial images. We mean by culture-centeredness the integration of people’s agency, voice, and power to direct community change through indigenous knowledge and ownership (Belone et al. 2016, 2017; Dutta 2007; Wallerstein et al. 2019). Research with AI/AN communities has increasingly turned to CBPR approaches to reduce disparities and strengthen wellness (Christopher et al. 2011; Dickerson et al. 2018; Ivanich et al. 2018). Tribal communities using CBPR strategies ensure that tribes are part of design, implementation, analysis, and dissemination of an innovation and can also enhance the capacity of implementers to transform the innovation for sustainability to their own settings (Dickerson et al. 2018; Walters et al. 2020).

### 1.3. Research and AI/AN Communities

Nationally, tribal oversight in research has grown in the past decades to counter historic abuses of “helicopter” research, stereotyping, and disregard for community knowledge. Tribes now demand the benefits of research studies, i.e., data ownership and authority over publications (Gittelsohn et al. 2020; Parker 2018). In the last decade, ethical guidelines for conducting research with AI/AN communities have recognized tribal sovereignty (NCAI Policy Research Center 2012; Straits et al. 2012; Walters et al. 2009). Shared principles include the right of native peoples to base research on their priorities, with longer timeframes, decolonized methodologies, and culture-centered interventions connected to history, land, and language (Bang et al. 2018; Drawson et al. 2017; Fisher and Ball 2003; Hallett et al. 2017; Simonds and Christopher 2013). While evidence demonstrates effectiveness of culturally specific interventions (IOM 2013; King et al. 2009; Mead et al. 2013), many still remain superficial with a focus on cultural images (i.e., feathers, arrows, dreamcatchers, etc.), rather than deeper cultural meanings and community self-determination (Kagawa Singer 2012; Kagawa-Singer et al. 2015).

CBPR has become a valued research approach between tribes and academic institutions as a counter to the history of research abuse and in recognition of the increasing evidence of CBPR effectiveness in reducing inequities (Jernigan et al. 2020a; Israel et al. 2013; Minkler et al. 2012; Sanchez-Youngman and Wallerstein 2018). Tribal participatory research, as a unique form of CBPR within AI/AN communities, has promoted the role of tribal sovereignty and leadership to direct the research, including IRB oversight (Fisher and Ball 2003; Parker 2018). Indigenous CBPR has also been adopted as a newer term indicating the importance of indigenous principles and decolonizing methodologies (Walters et al. 2020).

A recent special issue of *Prevention Science* of large-scale NIH-funded intervention trials, based on CBPR practices, for AI/AN communities has led to an upsurge of interest in the next step of D&I of interventions that are proving to be effective (Dickerson et al. 2018; Jernigan et al. 2020a; Jernigan et al. 2020b; Rasmus et al. 2019). The trials represent a range of origins, including adapting/re-centering trials from evidence from other populations or from culture-centered practices derived from communities or a mix. With D&I increasingly important in AI/AN communities, culturally appropriate frameworks are essential. Numerous frameworks support D&I efforts in non-AI/AN communities with 61 identified by one study (Tabak et al. 2012); yet, few have been tested with AI/AN research partnerships (Jernigan et al. 2020a).

Throughout the 15+ years of implementing the FLP and co-presenting at local and national conferences, the TRT/UNM-CPR team began receiving requests for information from multiple tribal communities interested in bringing the FLP to their community. We are reporting on two dissemination events that we held within the last year, first, a New Mexico (NM)-based mini-dissemination conference and, second, a pre-conference workshop for the 2019 National Indian Child Welfare Association (NICWA) conference. The goal of these events were to translate the research process into a program implementation process in establishing a FLP program with tribal communities who had shown an interest in re-centering and implementing their own FLP to be housed in their own health, education, or prevention departments.

The NM mini-conference came about through extensive planning by the TRT/UNM-CPR team who defined the conference structure, developed a recruitment plan, and scheduled a conference date. In the planning process, there was consensus that the sessions needed to have both interactive activities so that the participants could experience the FLP and information on the CBPR partnership between the TRTs and UNM-CPR to highlight the research development and implementation of the FLP in each community. Administration and program logistics were also key suggestions for the mini-conference based on the fact that each of the three TRTs had experienced important learnings within their own tribal administrative processes.

Participant recruitment to the NM mini-conference began in mid-summer 2018 with the posting of flyers in emails to tribal networks in New Mexico and to those who had previously indicated an interest in the FLP. The TRT/UNM-CPR team purposely chose to cover travel expenses (i.e., lodging, per diem, and mileage) of three individuals from six different tribal communities so that attendance by tribal members outside of Albuquerque did not become a burden for participating. The conference objectives were as follows: (1) to experience and gain knowledge about the family listening curriculum and determine feasibility for their community and (2) to gain an understanding of community-based participatory research processes for adapting and re-centering the program to their own tribal history, practices, knowledge, and values.

Based on the learnings and success of the NM mini-conference, the TRT/UNM-CRT team had a unique opportunity to share their dissemination experience through a pre-conference workshop prior to the National Indian Child Welfare Association (NICWA) conference that was to be held in Albuquerque in the spring of 2019. The TRT/UNM-CRT team reflected on their mini-conference experience and adjusted the agenda from a day-and-a-half conference to a successful six-hour workshop. These two dissemination experiences prompted the team to continue to work together and to develop an innovative framework that brings together the CBPR model, the Interactive Systems Framework.

### 1.4. Development of FLP Dissemination and Implementation of CBPR-CCS Framework

To create a D&I framework that would be appropriate for the multifaceted structure of tribal systems, the UNM research team identified the Interactive Systems Framework (ISF; Chambers 2012; Damschroder et al. 2009; Meyers et al. 2012; Wandersman et al. 2008). The ISF was identified as a D&I framework that would be congruent with our own CBPR culture-centered science (CBPR-CCS)_approach due to the following: accounting for the complexity of tribal systems’ oversight and approval of research and dissemination; being iterative in nature; recognizing the importance of practitioner knowledge for implementation in new settings; and supporting bi-directional learning, which is especially needed for cultural re-centering. The ISF therefore supports the critically important role of tribal sovereignty for approval, ownership, and sustainability. Our CBPR-CCS approach has evolved both from UNM’s Center of Participatory Research experience with the FLP (described above) and with our complementary investigation since 2006 to further the science of CBPR. We have asked the following: which partnering practices, under which contexts and conditions, translate to practice and health equity outcomes?

To strengthen the science of community engagement, our UNM research team, with national partners, has had three stages of NIH funding since 2006. We first developed and tested a CBPR conceptual model with four domains important in community–academy collaborations, which include the following: context; partnering processes and practices; processes and outputs of community-engaged research designs/interventions; and intermediate capacity and policy and longer-term health and health equity outcomes (Wallerstein et al. 2008; Belone et al. 2017; Kastelic et al. 2018). In the second stage, we identified measures from each domain of the CBPR model and conducted internet surveys of 200 diverse federally funded partnerships across the U.S., producing psychometrically validated scales of measures of partnering practices and outcomes, such as trust, participatory decision-making, and community engagement in all research stages, among others. (Oetzel et al. 2015). We analyzed associations and contributions of CBPR practices with outcomes, including the role of culture-centeredness (Duran et al. 2019; Oetzel et al. 2018; Wallerstein et al. 2019). We also conducted seven complementary case studies to further understand connections between practices and outcomes (Lucero et al. 2018). In our current third stage of funding, Engage for Equity (2015–2020), we refined measures and surveyed another 179 partnerships, validating psychometrics and associations between practices and outcomes, and have produced new workshops and tools for coaching partnerships to adopt promising practices (Wallerstein et al. 2020; Parker et al. 2020). Building trust has already been validated as an important promising practice with other analyses in development (Lucero et al. 2020). All funding stages had IRB approval from the University of New Mexico Health Sciences Center (currently: HRPO#16-098).

**Figure 1.**
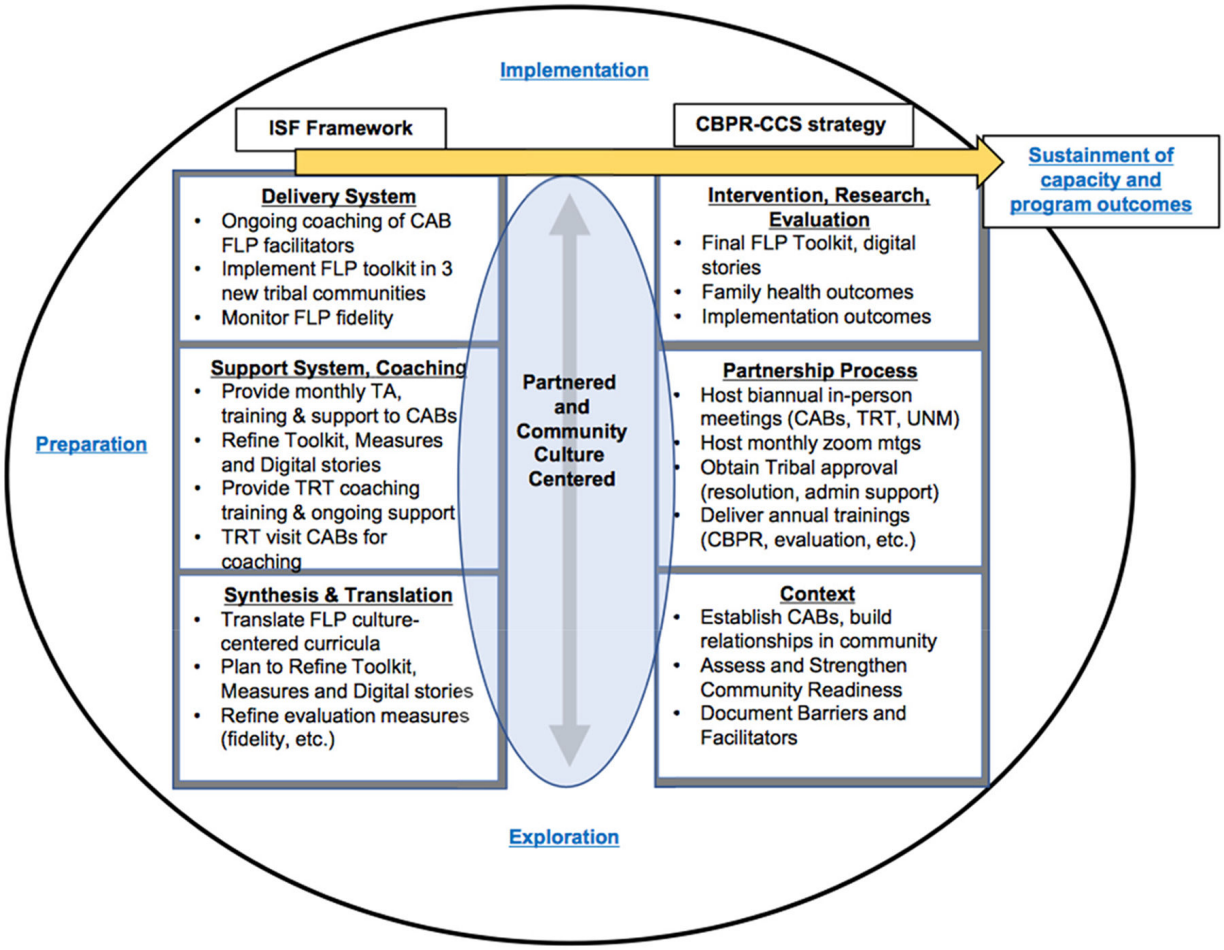
Community-based participatory research culture-centered science (CBPR-CCS)/Interactive Systems Framework (ISF) model.

For our FLP D&I approach, we combined our CBPR conceptual model, which includes a culture-centered approach to intervention development, with the ISF (Wandersman et al. 2008) to create a modified model framework (Figure 1) that incorporates CBPR and D&I processes. Our CBPR Culture-Centered Science (CBPR-CCS) framework accounts for the complexity of tribal systems’ oversight and approval of research and dissemination, is iterative in nature, and recognizes the importance of practitioner knowledge for implementation in new settings, supporting bi-directional learning, which is especially needed for cultural re-centering. Through its three systems, delivery, coaching, and translation, the ISF can also support the critically important role of tribal sovereignty for approval, ownership, and sustainability necessary for effective implementation among tribal communities.

## 2. Materials and Methods

The NM mini-dissemination conference was held on 6–7 September 2018 with 28 participants from six southwestern tribal communities, 10 more participants than anticipated with total attendance at 46, which included the TRT/UNM-CPR team. The participants were from the following communities: To’hajilee (the Cañoncito Band of Navajo), Taos Pueblo, Tesuque Pueblo, Kewa Pueblo, Zia Pueblo, and Navajo members of the San Juan Collaborative. The conference participants represented staff from education, behavioral health, language, prevention programs, and tribal leadership. Through breakout sessions, the TRT facilitators from Jemez Pueblo, Mescalero Apache, and Ramah Navajo led conference participants through four of the twelve sessions: (1) *Welcoming* (learning our Indian names and clans) and *Family and Community Values*; (2) *Our Family Tree*; (3) *Being Different*; and (4) *Our Tribal Histony*. In the breakout sessions, digital stories were shared that were produced by each TRT based on their own program, and in a discussion about the curriculum, a TRT facilitator stated, “The curriculum was developed by the community for the community, that’s why the program has been working for so long.”

While explaining the logistics about the program, a Mescalero Apache TRT member talked about the challenges and benefits of having support: “We were very fortunate to have research funding that helped us have transportation. We needed it because we had to pick up families and take them back after the program sessions.” Another important discussion focused on recruiting families and a Ramah Navajo TRT member shared, “we talk to the families and explain to them the program. We (also) go to the schools and talk to principals.” A Jemez Pueblo TRT member also added, “We have a newsletter that goes out monthly and explains how families can be recruited.” Lastly, in the creating your own family program session, the discussion centered on the importance of building a team and moving towards sustainability of the FLP program; a question was raised about procedures to start a program, and a Ramah Navajo TRT member responded that they identified another tribal department with similar goals, and this was helpful in the success of their FLP.

## 3. Results

Based on the success of the NM mini-conference, the TRT/ UNM-CPR team actively sought out the opportunity to host a pre-conference workshop at the 39th Annual NICWA, which was held in Albuquerque, NM, in March 2019. The workshop had 35 participants from across the U.S. and Canada who expressed interest in adopting the FLP. Based on the learnings from the NM mini-conference, the TRTs led participants through the same experiential hands-on FLP curriculum sessions focused on *Family Values, Being Different*, and *Tribal History*, as well as panels on community action projects, administration, and logistics for implementing the FLP. The challenge was modifying a day-and-a-half mini-conference into a six-hour workshop. Despite the shorter timeframe, the workshop successfully shared the FLP and garnered interest from several communities. One participant stated, “I will be talking with Tribal Council about applying or using this program.” Another participant shared that “I plan to relay this program to our Director. I will share the information I’ve gained to coworkers and I plan to use some of the concepts and icebreakers within our community.”

After experiencing these two dissemination conference/workshops, the TRT/UNM-CPR team turned these learnings into a NIH D&I grant proposal with the development of an innovative framework that brings together the CBPR-CCS model with the ISF (Figure 1). The ISF operationalizes three interacting systems (synthesis and translation, support, and delivery) that reciprocally bring practitioner knowledge into dialogue with the research innovation. The first stage, the *synthesis and translation system*, refines the evidence-based innovation into practice-based user-friendly processes, and CBPR-CCS incorporates contextual and cultural processes that enhance community readiness and capacity, based on the context domain of the CBPR model. The second ISF stage, *support system*, strengthens structural/functional factors (infrastructure, organizational capacity) and people power (capacity building, trainings, technical assistance) to ensure sufficient bi-directional knowledge for innovative uptake that leads to an improvement of the quality of the implementation. CBPR-CCS reinforces the support system with skilled-TRT members who will provide trainings and mentorship of the new tribal CABs through collaborative partnerships, relationship building, and navigation of complex tribal oversight and approvals that support tribal community-driven efforts. The third stage, the ISF *delivery system*, focuses on how the translated practice-based culture-centered *intervention and research*, the third domain of the CBPR model, will be implemented and evaluated for quality implementation to enable enhanced feasibility, cultural acceptance, with uptake and sustainment of new capacities and program outcomes as the final goal.

Surrounding this model are steps of implementation from the Exploration, Preparation, Implementation, and Sustainment (EPIS) framework (Aarons et al. 2011), helping us keep track of our grant steps. In sum, our integrated implementation strategy starts with the bottom right as the CBPR model context domain of assessing extent of collaboration and readiness matched with the bottom left opportunity to synthesize and culturally refine the specific intervention. Bi-directional partnership processes are then matched with the coaching support systems for implementation in other settings. Intervention and research actions are matched with how the intervention is delivered in the other settings. Finally, the integration leads to uptake and sustainability of capacities of new implementors as well as the potential for improved family outcomes.

After this integrated model was created, we adapted tools for evaluating how the FLP will be disseminated and implemented in other tribal communities. We integrated multiple qualitative and quantitative tools to assess implementation steps with the ISF coaching system (Wandersman et al. 2008) to increase our capacity to disseminate the FLP among tribal communities, first at the state level with plans for national dissemination. This included taking the existing FLP materials and data collection tools and incorporating a checklist of steps. In addition, process measures were identified to enhance the evaluation and implementation of the tools in the FLP Toolkit.

## 4. Discussion

In this paper, we describe the process of culturally centering the FLP and opportunities to improve D&I science through integrating a CBPR approach. The NM mini-dissemination conference and the NICWA pre-conference workshop were successful events that engaged participants from numerous tribal communities in deep and powerful discussions on the need for a tribally centered family intervention/program that could be re-centered in their own communities. Some highlights of the events, as expressed by the participants, were the presence and presentation by the different tribal communities (i.e., TRTs) and that the conference utilized positive experiential approaches and provided solutions (via the FLP program) to address community problems (i.e., CAPs). The participants commended the TRTs for their commitment to the creation and now sustainability of their FLPs in their communities and the long-term partnership between the TRTs and UNM-CPR. One of the participants praised the entire FLP team stating, “This work is not just bringing people together, but it is also making them aware about the issues to be addressed and why culture and language are important to be looked at.” Overall, the participants valued most the following topics: the process of curriculum re-creation, the involvement of language teachers, the role of facilitators and trainings, the administrative processes to run their own FLP, and the sharing of digital stories. Lastly, the participants not only learned about the FLP program, but were also able to network with plans to continue the dialogue about expanding the program to other tribal communities.

As a result of these two pilot D&I activities, we have strengthened our integrated D&I system, combining our CBPR- CCS model with ISF importance of providing tools and coaching for continued support to new tribal communities interested in the FLP. We also recognized the importance of capacity building of new FLP tribal teams, which will require ongoing consultation and coaching, especially in navigating support from tribal leadership and re-creating and re-centering the curriculum to their own culture, practices, history, and ways of knowing.

## 5. Conclusions

In this paper, we shared the pilot dissemination efforts of the culture-centered FLP, an intergenerational indigenous family program based on a long-standing CBPR partnership between three research teams from three American Indian communities and UNM-CPR. The findings from the pilot dissemination activities moved the TRT/UNM-CPR team to developed an innovative D&I model, which integrated CBPR-CCS with the ISF; this process will strengthen D&I science by testing the implementation of our indigenous-focused FLP with implications for indigenous global health. Future research should continue to examine appropriate D&I strategies among indigenous communities to improve health disparities.
